# Cucurbitacins: elucidation of their interactions with the cytoskeleton

**DOI:** 10.7717/peerj.3357

**Published:** 2017-05-30

**Authors:** Xiaojuan Wang, Mine Tanaka, Herbenya Silva Peixoto, Michael Wink

**Affiliations:** Institute of Pharmacy and Molecular Biotechnology, Ruprecht-Karls-Universität Heidelberg, Heidelberg, Germany

**Keywords:** Cucurbitacins, Cucurbitaceae, Cytotoxicity, Actin filament, Microtubule

## Abstract

Cucurbitacins, a class of toxic tetracyclic triterpenoids in Cucurbitaceae, modulate many molecular targets. Here we investigated the interactions of cucurbitacin B, E and I with cytoskeletal proteins such as microtubule and actin filaments. The effects of cucurbitacin B, E and I on microtubules and actin filaments were studied in living cells (Hela and U2OS) and *in vitro* using GFP markers, immunofluorescence staining and *in vitro* tubulin polymerization assay. Cucurbitacin B, E and I apparently affected microtubule structures in living cells and cucurbitacin E inhibited tubulin polymerization *in vitro* with IC_50_ value of 566.91 ± 113.5 µM. Cucurbitacin E did not affect the nucleation but inhibited the growth phase and steady state during microtubule assembly *in vitro*. In addition, cucurbitacin B, E and I all altered mitotic spindles and induced the cell cycle arrest at G2/M phase. Moreover, they all showed potent effects on actin cytoskeleton by affecting actin filaments through the depolymerization and aggregation. The interactions of cucubitacin B, E and I with microtubules and actin filaments present new insights into their modes of action.

## Introduction

**Figure 1:**
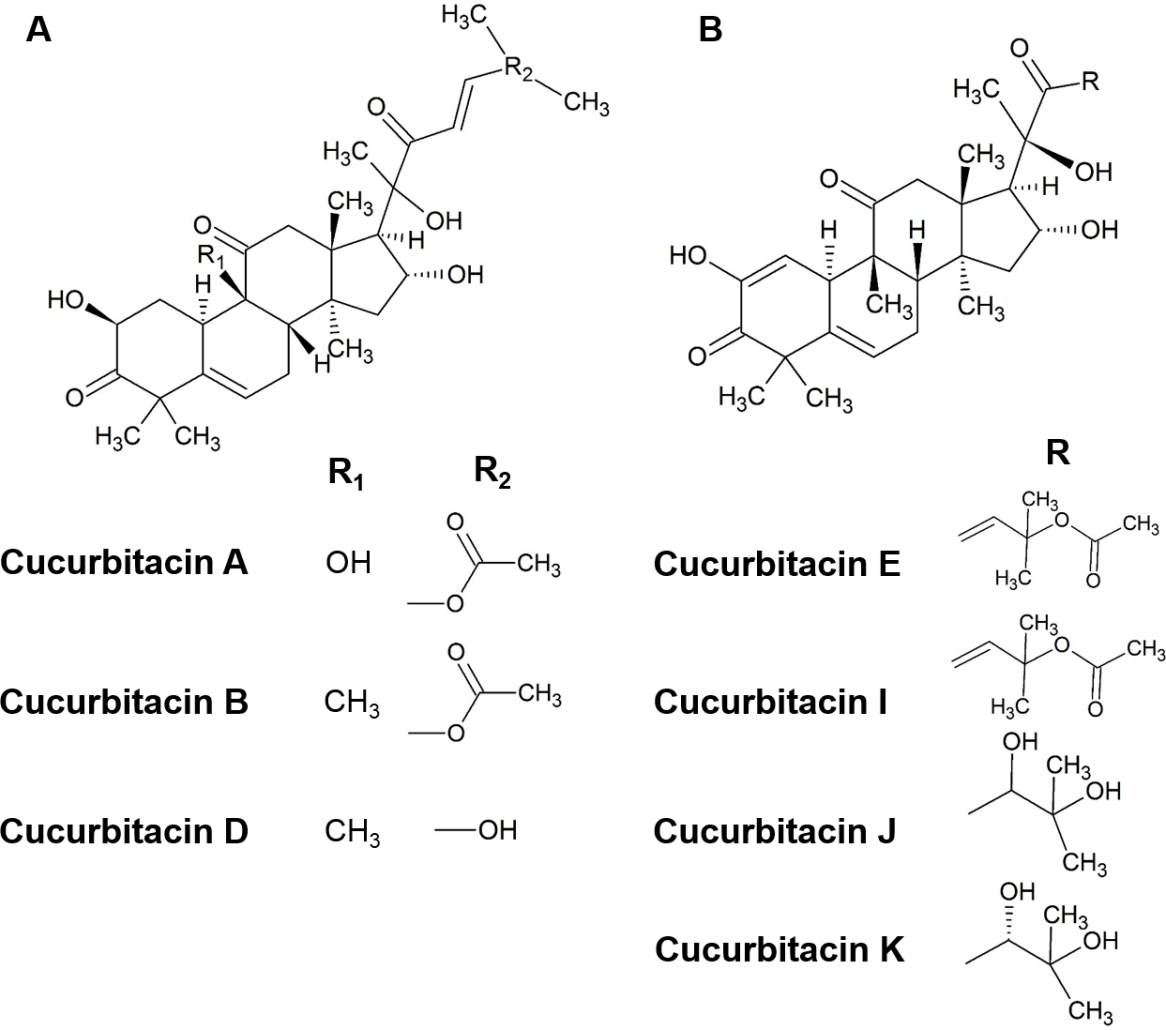
The structure of cucurbitacins. (A) The skeleton of cucurbitacin A, B and D. (B) The skeleton of cucurbitacin E, I, J and K.

Cucurbitacins are a class of cucurbitane-type tetracyclic triterpenoids that are mainly produced by plants of the family of Cucurbitaceae (Duncan et al., 1996; Kaushik, Aeri & Mir, 2015; Wink & Van Wyk, 2008). Hundreds of cucurbitacins that occur in a diversity of plants share the same tetracyclic scaffold and can be divided into 12 main categories according to their substituents (Chen et al., 2012; Lee, Iwanski & Thoennissen, 2010). Cucurbitacins B and E (Fig. 1) have been identified to be the primary cucurbitacin types by plant secondary metabolism studies (Abbas et al., 2013; Gry, Søborg & Andersson, 2006; Kaushik, Aeri & Mir, 2015). Under certain environmental conditions, other cucurbitacin types could be generated by enzymatic reactions. For instance, cucurbitacins A, C, D can be produced from cucurbitacin B, while cucurbitacins I, J, K can be generated from cucurbitacin E (Ahmed & Halaweish, 2014; Chen et al., 2012).

Cucurbitacins exhibit a broad range of pharmacological properties such as anti-inflammatory, antioxidant, antiviral, antipyretic, analgesic and anti-malaria activities (Chen et al., 2005; Jayaprakasam, Seeram & Nair, 2003; Miro, 1995). Current studies have revealed several molecular targets of cucurbitacins such as JAK2/STAT3 pathway, cofilin, cyclins, cdc2, COX-2, TYR and EcR, among which actin cytoskeleton appears to be an early target (Blaskovich et al., 2003; Chen et al., 2012). Additionally, cucurbitacin B has been reported to disrupt microtubule polymerization in several studies (Yin et al., 2008; Duangmano et al., 2012). However, only few studies have explored the effects of cucurbitacins on the microtubule-based cytoskeleton and the underlying mechanisms of action of cucurbitacins remains elusive.

Actin filaments and microtubules, the two major networks of the eukaryotic cell cytoskeleton, become attractive targets for natural compounds in cancer research due to their importance in a board range of processes such as vesicular and organelle transport, cell proliferation and migration (Jordan & Wilson, 2004; Petrasek & Schwarzerova, 2009; Wickstead & Gull, 2011). In this study, we investigated the effects of cucurbitacin B, E and I on microtubules and actin filaments in living cells (cancer cell lines Hela, MCF7, and U2OS) using GFP markers and immunofluorescence staining. Their interactions with tubulin dynamics were further determined *in vitro* using tubulin polymerization assay. Reference drugs such as colchicine and vinblastine (microtubule-binding agents) and latrunculin B (actin-binding agent) were used as comparing controls. We can provide evidence for unidentified interactions between cucurbitacins and the cytoskeleton in this study.

## Materials and Methods

### Cell lines, chemicals and laboratory materials

The human cervical cancer cell line Hela was purchased from ATCC (Wesel, Germany) and the MCF-7 human breast cancer cell line was provided by Prof. Dr. Stefan Wölfl (Institute of Pharmacy and Molecular Biotechnology, Heidelberg University, Heidelberg, Germany); U2OS human osteosarcoma cancer cells which were stably transfected with α-tubulin-GFP construct were supplied by Prof. Dr. Thomas Efferth (Institute of Pharmacy and Biochemistry, Johannes Gutenberg University, Mainz, Germany); cucurbitacin E and I (purity > 99% by HPLC) came from Phytoplan GmbH (Heidelberg, Germany) and cucurbitacin B (purity > 98% by HPLC) from Baoji Herbest Bio-Tech Co., Ltd. (Baoji, Shannxi, China); vinblastine (1 mg/mL) were purchased from Central Pharmacy of the University Hospital Heidelberg (Heidelberg, Germany); colchicine (purity > 95% by HPLC), latrunculin B (purity > 80% by HPLC), G418, Atto 390 phalloidin, paraformaldehyde, propidium iodide, ATP, BSA, Dimethyl sulfoxide (DMSO), EDTA, EGTA, FBS, GTP, MTT, piperazine-N, N′-bis(2-ethanesulfonic acid) (PIPES), RNase A and Coomasie blue were obtained from Sigma-Aldrich Chemie GmbH (Steinheim, Germany) and mowiol 4–88 from Carl Roth GmbH & Co. KG (Karlsruhe, Germany); DMEM, non-essential amino acids, penicillin-streptomycin, CellLight^®^ Actin-RFP BacMam 2.0 actin-RFP, trypsin-EDTA came from Life technologies (Paisley, United Kingdom) and triton X-100 from Merck KgaA (Darmstadt, Germany); mouse anti-α-tubulin monoclonal IgG and goat anti-mouse IgM-FITC were obtained from Santa Cruz Biotechnology (Heidelberg, Germany); 96-well-plates, 24-well-plates and 6-well-plates were purchased from Greiner (Frickenhausen, Germany) and circular glass coverslips from Thermo Scientific (Braunschweig, Germany).

### Cell culture

Hela, MCF-7 and U2OS cancer cells were cultivated as previously described (Wang et al., 2016b).

### MTT assay

The anti-proliferative effects of cucurbitacins were assessed using MTT assay, as previously described (Nurcahyanti & Wink, 2015). In brief, cells (1 × 10^4^) were seeded in 96-well plates and incubated with different concentrations of cucurbitacins for 48 h (Hela, U2OS) and 72 h (MCF-7). MTT solution was then added and incubated for 2 h. The plates were read at 570 nm after the addition of DMSO using Tecan infinite M200 Pro (Tecan, Crailsheim, Germany).

### Imaging of tubulin-GFP transfected U2OS cells

α-Tubulin-GFP U2OS cells (1 × 10^5^) were seeded in 24-well-plates and treated with 200μl different concentrations (IC_80_, IC_50_ based on MTT data) of cucurbitacins. Cells were imaged using a Keyence BZ-9000 microscope (Keyence; Neu-Isenburg, Germany) after incubation for 2 h, 4 h, 24 h and 48 h. The images were analyzed using BZ-II Analyzer software (version 2.1, Keyence; Neu-Isenburg, Germany).

### Immunofluorescence staining

The immunofluorescence staining was carried out as established in our laboratory (Wang et al., 2016a).

### Imaging of actin-RFP transfected hela cells

2 × 10^4^ Hela cells were seeded in 24-well-plates and mixed with CellLight^®^ Actin-RFP BacMam 2.0 which is a fusion construct of human actin and TagRFP, providing an accurate and specific targeting to cellular actin filaments. After 16 h of incubation, 200 μl different concentrations of cucurbitacins (IC_80_, IC_50_ based on MTT data) were added and the cells were analyzed as described above (‘Imaging of tubulin-GFP transfected U2OS cells’).

### *In vitro* tubulin polymerization assay

Porcine brain tubulin plus MAPs was prepared by two cycles of polymerization and depolymerization according to a standard protocol (Gell et al., 2011). *In-vitro* tubulin polymerization assays were carried out in PEM buffer (100 mM PIPES, 2 mM EGTA, 0.1 mM EDTA, 3 mM MgCl_2_, 1 mM ATP and 1 mM GTP, pH 6.85) by mixing 5.6 mg/ml tubulin-MAPs with different concentrations of cucurbitacins in 96-well plates at 37°C for 40 min. The rate and extent of the polymerization reaction were monitored by light scattering at 360 nm using Tecan infinite M200 Pro.

### Cell cycle analysis

Cell cycle analysis was carried out as established in our laboratory (Su, Cheng & Wink, 2015). Briefly, Hela cells (5 × 10^5^) were seeded in 6-well-plates and treated with different concentrations of cucurbitacins for 24 h. Cells were then collected, centrifuged and fixed in 70% ice-cold ethanol for at least 8 h. After washing steps, cells were treated with 0.2 mg/ml RNase A for 30 min at 37 C and then stained with 0.1 mg/ml propidium iodide. Samples were analyzed using a FACScan flow cytometer (Becton Dickinson, Heidelberg, Germany). Data were analyzed using Cell Quest™ Pro software (Becton Dickinson) and Microsoft excel (Microsoft Corporation, Washington, USA).

### Statistical analysis

The data of reference drugs colchicine, vinblastine and latrunculin B have been published before by us Wang et al. (2016a). The IC_50_ and IC_80_ were determined as the amount of the substances needed to reduce 50% or 80% cell viability/tubulin polymerization and calculated from concentration–response curves by Sigmaplot software (Systat Software Inc., San Jose, USA). All experiments were done in triplicate, repeated three times. Data are presented as mean ± standard deviation (SD). Statistical comparison between controls and different treatments were performed by an unpaired student’s *t* test using Microsoft excel 2013 (Microsoft Corporation, Washington, USA). Significance was considered at *p* < 0.05.

## Results

### Cytotoxicity of cucurbitacins

The anti-proliferative activities of cucurbitacins against Hela, MCF-7 and U2OS cells were assessed by MTT assay (Table 1). Among the reference drugs, the known actin-binding agent latrunculin B inhibited the growth of three cell lines with IC_50_ values between 5.67 μM and 38.5 μM. Compared to latrunculin B, cucurbitacin B, E and I exhibited stronger cytotoxicity against all three cell lines with IC_50_ values between 6.43 nM and 64.67 nM. The known microtubule-binding agent colchicine and vinblastine also showed stronger anti-proliferative activity than latrunculin B with IC_50_ values between 0.02 nM and 30.29 nM. Compared to colchicine and vinblastine, cucurbitacin B, E and I exhibited lower IC_80_ values (15.09 nM–0.92 μM) but greater IC_50_ values (6.43 nM–64.67 nM). Among these cucurbitacins, cucurbitacin B and E caused a higher toxicity than cucurbitacin I, which is close to the microtubule-binding agent colchicine.

**Table 1:** Cytotoxic activities of cucurbitacins and reference drugs against Hela, MCF-7 and U2OS cells.

Compounds	IC_80_			IC_50_		
	Hela	MCF-7	U2OS	Hela	MCF-7	U2OS
Colchicine^a^	27.01 ± 7.48 nM	79.89 ± 40.85 nM	0.87 ± 1.46μM	14.9 ± 3.94 nM	30.29 ± 8.02 nM	25.2 ± 19.58 nM
Vinblastine^a^	0.7 ± 0.34μM	2.08 ± 0.92μM	1.15 ± 0.51μM	0.02 ± 0.01 nM	0.06 ± 0.05 nM	0.11 ± 0.07 nM
Latrunculin B^b^	63.94 ± 5.68μM	140.1 ±6.58μM	37.17 ± 15.68μM	11.19 ±1.27μM	38.5 ± 1.7μM	5.67 ± 0.59μM
Cucurbitacin B	22 ± 1.39 nM	43.71 ± 10.61 nM	28.05 ± 15.25 nM	12.2 ± 1.42 nM	22.93 ± 4.51 nM	17.07 ± 4.55 nM
Cucurbitacin E	15.09 ± 2.67 nM	0.92 ± 0.2μM	26.27 ± 18.50 nM	6.43 ± 1.05 nM	54 ± 3.16 nM	15.07 ± 4.51 nM
Cucurbitacin I	55.49 ± 3.63 nM	0.29 ± 0.08μM	34.03 ± 17.74 nM	44.77 ± 1.54 nM	64.67 ± 14.29 nM	23.47 ± 16.92 nM

**Notes.**

a Active on tubulin/microtubules.

b Active against actin filaments; data are presented as mean ± SD.

### Cucurbitacins interfered with microtubule structures in living cells

#### Influence on microtubules

**Figure 2:**
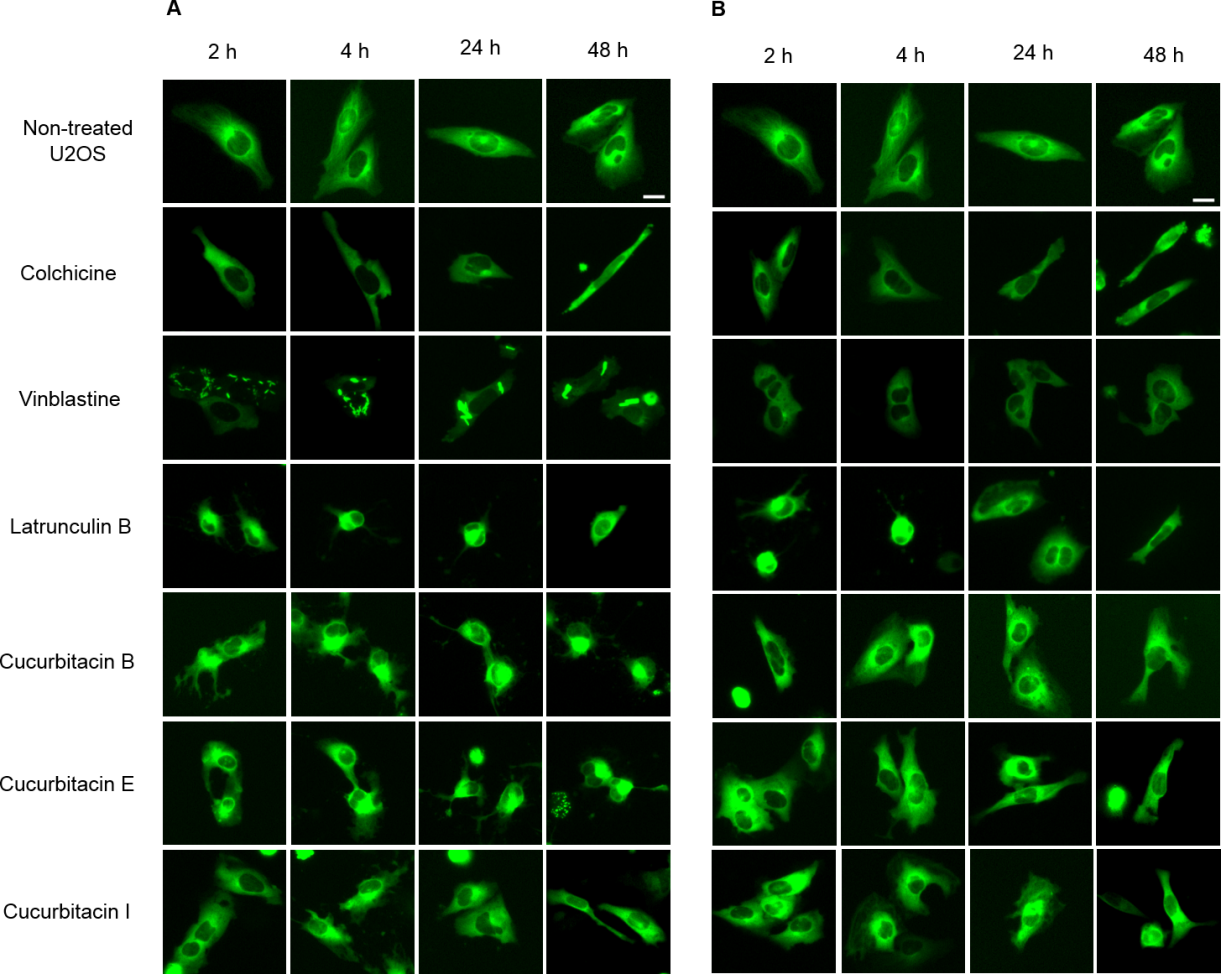
Cucurbitacins changed the morphology of microtubule network in U2OS cells. Panels show micrographs of U2OS cells treated for 2 h, 4 h, 24 h and 48 h with all six compounds at concentrations of IC_80_ (A) and IC_50_ (B). Known tubulin inhibitors colchicine and vinblastine induced microtubule depolymerization and tubulin paracrystals, respectively. Actin-binding agent latrunculin B caused the rapid change of cell morphology. Bar = 10 µm.

The U2OS cells which express α-tubulin-GFP were treated with cucurbitacins to determine their effects on the cellular microtubule network by observing the changes in living cells (Fig. 2). In non-treated cells, microtubules extended continuously through the cytoplasm and formed an extensive intracellular network. Treatment with colchicine at both concentrations (IC_80_, IC_50_) decreased the microtubule mass, which exhibited a reduced intensity at the cell periphery compared to non-treated cells. Vinblastine depolymerized microtubules in a way different from colchicine that tubulin paracrystals were formed and dispersed through out the cytoplasm at the concentration of IC_80_. While at the concentration of IC_50_, tubulin paracrystals disappeared and extensively depolymerized microtubules were observed. Latrunculin B immediately changed the cell morphology from stretching state into round state at both concentrations (IC_80_, IC_50_), which was returned to normal morphology after 24 h incubation with the microtubule mass slightly decreased. The effect of cucurbitacins on microtubule network was concentration-dependent and different from other reference drugs. Cucurbitacin B and E firstly changed the morphology of microtubule network into half-stretching state after 2 h incubation then round state after 24 h incubation, which exhibited their significant interference on microtubule network. Cucurbitacin I also induced the similar but weaker effect on microtubule network after 4 h incubation.

#### Influence on spindle apparatus

**Figure 3:**
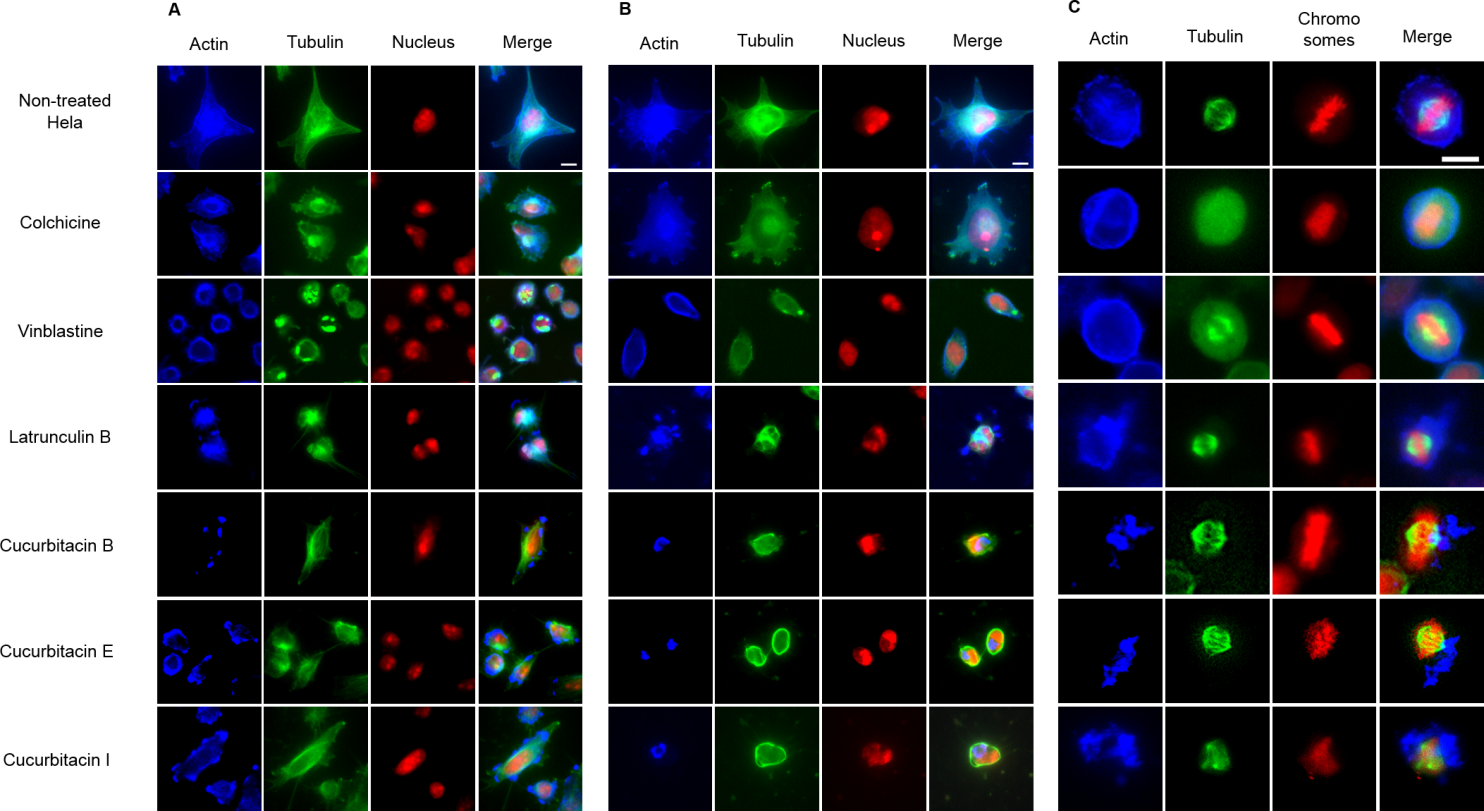
The effects of cucurbitacins on Hela mitotic cells using immunofluorescence staining. Microtubules & mitotic spindles were stained by mouse anti-*α*-tubulin monoclonal IgG and goat anti-mouse IgM-FITC (green), actin filaments were stained by Atto 390 phalloidin (blue) and nucleus & chromosomes were stained by propidium iodide (red). (A) and (B) show immunofluorescence micrographs of Hela interphase cells treated for 1 h and 24 h with all six compounds at the concentration of IC_80_. Known tubulin inhibitors colchicine and vinblastine induced microtubule depolymerization and tubulin paracrystals, respectively. Actin-binding agent latrunculin B caused the rapid change of cell morphology and depolymerization of actin filaments. Cucurbitacins changed the morphology of microtubule network and caused actin aggregation. (C) shows immunofluorescence micrographs of Hela metaphase cells treated for 24 h with all six compounds at the concentration of IC_50_. Absent spindle and depolymerized bipolar spindles were caused by colchicine and vinblastine, respectively. Latrunculin B depolymerized actin filaments without alternating spindles and chromosomes arrangement. Cucurbitacins altered mitotic spindles and induced actin depolymerization and aggregation. Bar = 10 µm.

The effects of cucurbitacins on mitotic microtubules were further evaluated by immunofluorescence staining in Hela cells (Fig. 3). The effects of colchicine, vinblastine, latrunculin B and cucurbitacins on Hela interphase microtubule network (Figs. 3A and 3B) were comparable with the findings in U2OS cells. In non-treated Hela metaphase cells (Fig. 3C), microtubules formed symmetric bipolar spindles with chromosomes aligning at the metaphase plate. The completely depolymerized spindle with the compacted chromosomes were observed in colchicine-treated cells, while depolymerized bipolar spindles were found in vinblastine-treated cells. Latrunculin B did not alter mitotic spindles and chromosome arrangements in Hela cells. Cucurbitacin B and E caused disordered distribution of spindle array, while cucurbitacin I led to multipolar spindles and chromosomes mis-segregation on the metaphase plate.

### Cucurbitacin E inhibited tubulin polymerization *in vitro*

**Figure 4:**
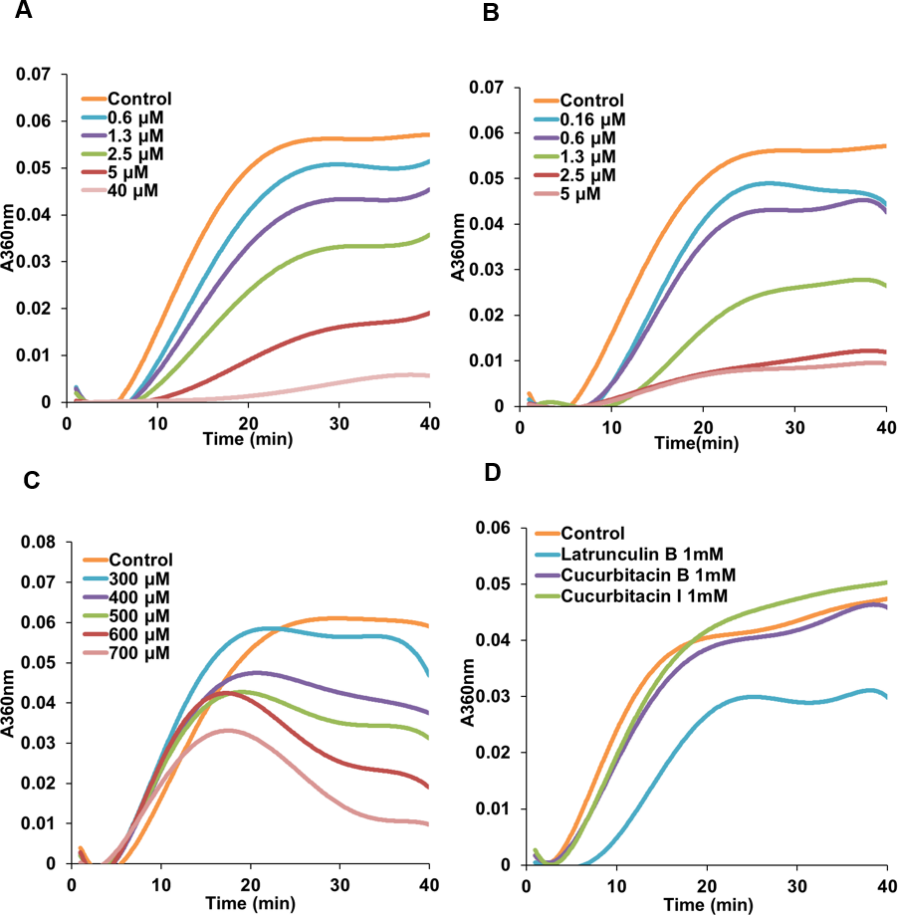
Cucurbitacin E inhibited tubulin polymerization *in vitro*. Polymerization of tubulin with MAPs in the assembly buffer was measured in the absence (⧫) and in the presence of different concentrations of compounds. (A), (B) Colchicine and vinblastine inhibited the nucleation and growth phase during the assembly. (C) Cucurbitacin E did not affect the nucleation but inhibited the growth phase and steady state. (D) Cucurbitacin B and I did not affect tubulin polymerization and latrunculin B showed weak inhibition on the dynamic.

Due to the initial results (Figs. 2 and 3) which indicated a potential interference of cucurbitacins with microtubules, the direct effects of cucurbitacins on the assembly of tubulin into microtubules were determined *in vitro* (Table 2). However, only cucurbitacin E exhibited a direct but weak inhibition on tubulin polymerization with IC_50_ value of 566.91 ±113.5μM, while cucurbitacin B and I did not affect the tubulin assembly *in vitro*. Known tubulin inhibitors colchicine and vinblastine showed a more pronounced inhibition on tubulin assembly with IC_50_ values of 2.86 ±0.16μM and 1.57 ±0.34μM, respectively. Actin-binding agent latrunculin B did not exhibit significant inhibition on tubulin assembly. Figure 4 illustrates the tubulin polymerization dynamics of each compound. In the absence of compounds, the assembly of tubulin into microtubules begins with a slow formation of the microtubule nucleus, which is followed by the rapid elongation of the nucleus polymer. When the growth of one end balances the shrinkage of the other end on the polymer, the polymerization dynamic reaches steady state (Grintsevich & Reisler, 2013; Margolis & Wilson, 1998). The effects of colchicine and vinblastine on tubulin assembly were similar: As the concentration increased, the time needed for nucleation was longer and the growth phase of microtubule polymer was shorter, which led the system to the equilibrium phase sooner. However, the mode of action of cucurbitacin E was different that it did not affect the nucleation but inhibited the growth phase and steady state. Figure 4D showed that 1 mM cucurbitacin B and I did not affect the assembly, while 1 mM latrunculin B weakly inhibited the polymerization around 30%.

**Table 2:** Inhibition of tubulin polymerization *in vitro*.

Compounds	IC_50_
Colchicine^a^	2.86 ± 0.16μM
Vinblastine^a^	1.57 ± 0.34μM
Latrunculin B^b^	>1 mM
Cucurbitacin B	>1 mM
Cucurbitacin E	566.91 ± 113.5μM
Cucurbitacin I	>1 mM

**Notes.**

a Active on tubulin/microtubules.

b Active against actin filaments; data are presented as mean ± SD.

### Cucurbitacins exhibited dramatic effects on actin filaments

#### Influence on mitotic actin filaments

The effects of cucurbitacins on actin filaments were firstly evaluated by immunofluorescence staining in Hela mitotic cells (Fig. 3). The actin-binding agent Latrunculin B significantly altered the cell shape after 1 h incubation, which partially recovered after 24 h with the actin cytoskeleton extensively disrupted. Latrunculin B also affected metaphase cells by depolymerizing the actin filaments without alternating spindles and chromosomes arrangement. No apparent changes on actin filaments were found in colchicine-treated cells. In vinblastine-treated cells, a slight reduction of actin filament mass was observed after 24 h incubation at the concentration of IC_80_ (Fig. 3B). Cucurbitacins exhibited remarkable effects on actin filaments both in Hela interphase and metaphase cells: after 1 h treatment, the actin network started to depolymerize and the cell shape was slightly changed (Fig. 3A): After 24 h incubation, the cell morphology was dramatically deformed and the aggregation of actin filaments into one piece was observed (Fig. 3B); in metaphase cells, actin depolymerization and aggregation were greatly accentuated (Fig. 3C).

#### Influence on cellular actin filaments

**Figure 5:**
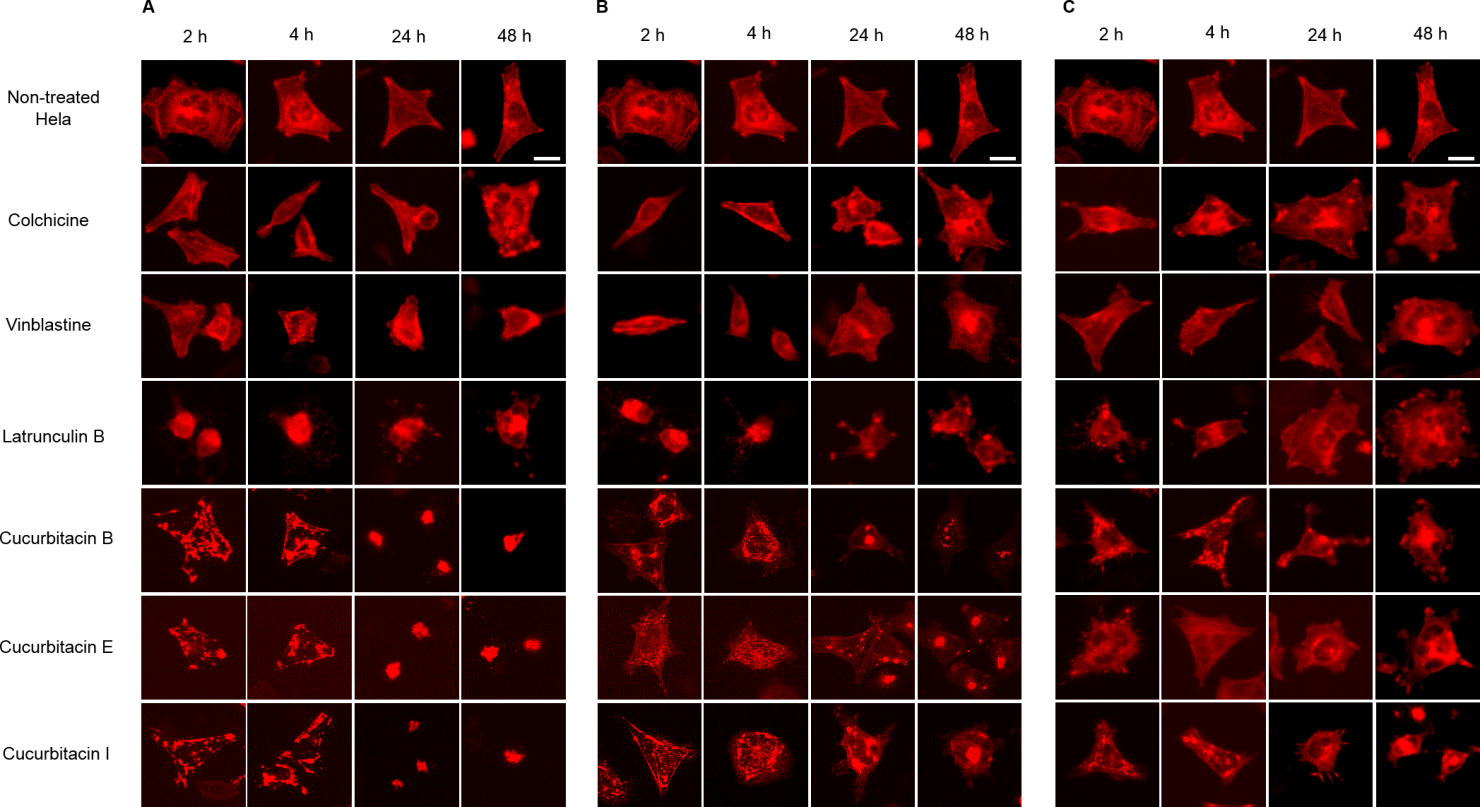
Cucurbitacins changed the cell morphology and reduced the mass of actin filaments after 24 h treatment. Panels show micrographs of Hela cells which were transduced with actin-RFP treated for 2 h, 4 h, 24 h and 48 h with all six compounds at concentrations of IC_80_ (A), IC_50_ (B) and IC_20_ (C). Actin-binding agent latruculin B induced the change of cell morphology and extensive depolymerization of actin network. Colchicine caused few changes on actin network and vinblastine slightly reduced actin filament mass after 4 h incubation at high concentration of IC_80_. Bar = 10 µm.

Hela cellular actin filaments were further visualized by actin-RFP and treated with cucurbitacins to evaluate their effects on cellular actin filaments in living cells (Fig. 5). The results were in agreement with the findings shown in Fig. 3. No notable changes on actin filaments were observed in colchicine/vinblastine-treated cells. The actin-binding agent Latrunculin B immediately altered the cell shape after 2 h incubation, which partially recovered after 24 h with the actin cytoskeleton significantly disrupted. Cucurbitacins acted differently from latruculin B: At the concentration of IC_80_, the actin network was extensively disrupted within 24 h incubation and granulated aggregations of condensed actin were dispersed through out the cytoplasm; After 24 h incubation, the cell morphology started to change, while actin aggregation accentuated and tented to gather into one instead of distributing through out the whole cell (Fig. 5A). These effects were weakened as cucurbitacins concentration decreased (Fig. 5B), but slight aggregation of actin still can be observed at the concentration of IC_20_ (Fig. 5C). These results suggest that cucurbitacins remarkably rearrange actin cytoskeleton and their mechanism of action is different from that of latrunculin B.

### Cucurbitacins arrested cell cycle at G2/M phase

**Figure 6:**
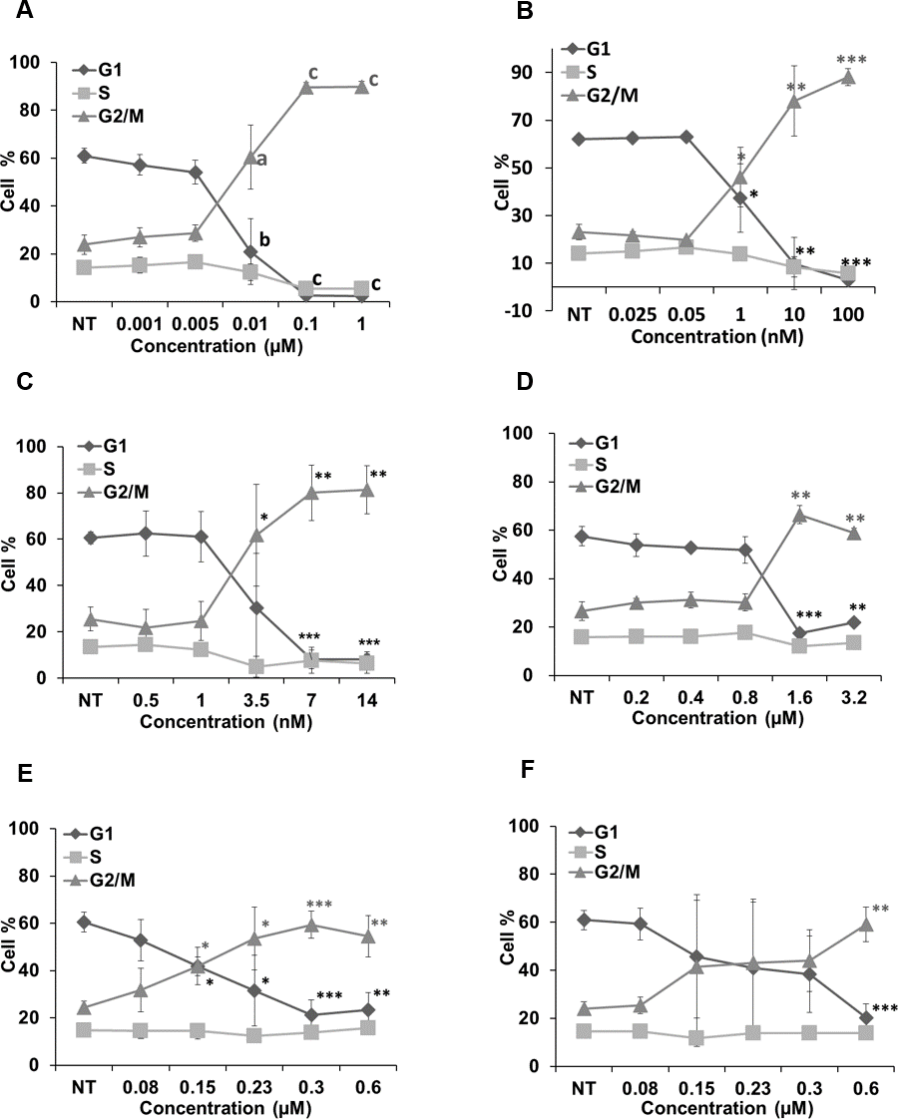
Cell cycle analysis in Hela cells. Cells were harvested after 24 h of drug treatment and subsequently assayed for their DNA content by flow cytometry. Colchicine, vinblastine, latrunculin B and cucurbitacins all blocked cell cycle at G2/M phase (A–F). Data are represented as mean ± SD from three independent experiments. ^∗^*p* < 0.05, ^∗∗^*p* < 0.01, ^∗∗∗^*p* < 0.001.

Figure 6 represents the effects of cucurbitacins on cell cycle. Colchicine, vinblastine, latrunculin B and cucurbitacins all induced a dose-dependent G2/M cell cycle arrest. Colchicine, vinblastine and latrunculin B exhibited stronger effects on cell cycle than cucurbitacins. Colchicine and vinblastine promoted the G2/M population to 89.66 ± 2.04% (*p* < 0.001) and 78.04 ± 14.78% (*p* < 0.01) at the concentration of 0.1μM and 10 nM, respectively. While cucurbitacin B, E and I promoted the G2/M population to 66.41 ± 3.73% (*p* < 0.01), 59.35 ± 5.69% (*p* < 0.001) and 59.18 ± 7.2% (*p* < 0.01) at the concentration of 1.6μM, 0.3μM and 0.6μM, respectively. These results indicate the potential ability of cucurbitacins to act as antimitotic agents.

## Discussion

In this study, we provide evidence that cucurbitacin B, E and I interacted with actin filaments through the induction of aggregation and depolymerization in Hela and U2OS cells, which allows a more comprehensive understanding of the changes of actin filaments in cancer cells responding to cucurbitacins. In addition, they also interfered with microtubule structure and altered mitotic spindles in living cells though their effects on tubulin polymerization are weak.

The cytotoxicity of each cucurbitacin was similar on Hela, MCF-7 and U2OS cells (Table 1), indicating cucurbitacins do not have an apparent specific toxicity on cancer cell lines. Cucurbitacins exhibited strong anti-proliferative activities against the three cell lines, among which cucurbitacin B and E showed the similar cytotoxicity as colchicine, suggesting their potential applications on cancer treatment. Cucurbitacins contain a Michael acceptor ( 
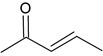
 ) at the side chain and a hydroxyl group at C3, which have been revealed to play important roles in their cytotoxicity via structure–activity relationship study (Chen et al., 2012; Duncan et al., 1996). Additionally, based on our data, cucurbitacin B and E caused a higher toxicity than cucurbitacin I, suggesting the –OAc group at the side chain may also contribute to their cytotoxic properties.

The cytotoxicity of cucurbitacins is most likely correlated with their actions on actin filaments. In this work, we found that cucurbitacins rearranged actin cytoskeleton even at low concentration (IC_20_, Fig. 5C), indicating that cucurbitacins affect actin filaments with high affinity and may induce apoptosis mainly through the disruption on actin filaments. The mode of action of cucurbitacins on actin filaments was different from that of latrunculin B in that the actin network was extensively depolymerized and the disrupted filaments were condensed and aggregated (Fig. 5). Cucurbitacins have been reported to covalently bind to cofilin which is an actin-binding protein, resulting in the increase of actin depolymerization (Gabrielsen et al., 2013; Lappalainen & Drubin, 1997; McGough et al., 1997; Nakashima et al., 2010). Interestingly, Sari-Hassoun et al. (2016) recently found that cucurbitacin I does not bind to cofilin; instead, it is a direct inhibitor of LIMK1, a kinase that regulates the phosphorylation of cofilin. Although these hypotheses are controversial, one still could speculate that in cucurbitacin-treated cells, the pathways involved in cofilin activation are related to the actin depolymerization caused by cucurbitacins. However, the actin network was not only severed but also condensed in cucurbitacin-treated cells. Phalloidin and jasplakinolide are actin-stabilizing agents that inhibit depolymerization and stabilize the structure of actin filament (Cooper, 1987; Holzinger, 2009). Cucurbitacins have been reported to substoichiometrically bind to actin and stabilize the polymerized actin without affecting its assembly (Momma et al., 2008; Sorensen et al., 2012). Cucurbitacins do not compete with phalloidin and jasplakinolide for the same binding site, which reveals that their mechanisms of action are different from phalloidin and jasplakinolide (Sorensen et al., 2012). Zhang et al. (2014) suggest that the actin aggregation induced by cucurbitacin B is mediated via G α13/RhoA/PKA/VASP pathway. While Sari-Hassoun et al. (2016) suggest the aggregation of actin induced by cucurbitacin I most probably results from the stimulation of the Rho/ROCK pathway. Another less probable proposal is that cucurbitacins may sever and stabilize the actin via the modifications of its cysteines, since the Michael acceptor of cucurbitacins can react with -SH protein by forming a covalent bond (Gabrielsen et al., 2013; Kumar et al., 2016; Sorensen et al., 2012). The effects of cucurbitacins on the actin cytoskeleton have been observed two decades ago; however, the precise mechanism is still not fully or correctly understood and more work is needed.

Furthermore, we discovered that cucurbitacins significantly interfered with microtubule structure and altered mitotic spindles in cells (Figs. 2 and 3), which indicates the new relationship between cucurbitacins and microtubules. However, the *in vitro* tubulin polymerization assay showed that except cucurbitacin B and I, only cucurbitacin E exhibited a direct but weak inhibition on the assembly (Table 2), indicating that the –OAc group at side chain and the ring may be involved in the interaction between cucurbitacin E and tubulin assembly. Duangmano et al. (2012) also observed that cucurbitacin B did not affect tubulin polymerization *in vitro* using the same assay. Taken together, it can be assumed that cucurbitacins act on cellular microtubules not mainly by the direct interaction with tubulin, but by the indirect effects. Through to the above findings, the effects of cucurbitacins on microtubules and actin filaments may throw up the questions that did these effects correlate with each other and was one effect the cause of the other? Microtubules and actin filaments cooperate functionally in a board range of processes, including vesicular and organelle transport, cell and nuclear migration, spindle rotation and cleavage furrow placement via a series of accessory proteins such as kinesin, myosin, dynein, Anillin, RacGAP50C etc. (D’Avino et al., 2008; Goode, Drubin & Barnes, 2000). Microtubule-binding agents such as colchicine and vinblastine which bound to tubulin subunit and depolymerized microtubules, did not affect actin filaments (Figs. 2–5), indicating the direct alteration to microtubules does not directly affect actin filaments. Thus, it highly suggests that the interaction with microtubules would not lead to the alteration on actin fialments. On the other hand, though cucurbitacins significantly affected actin filaments, their effects on microtubules were indirect and there is no relevant evidence to demonstrate the relationship between cucurbitacins and those accessory proteins. Thus, we hardly make the conclusion that the alteration on actin filaments by cucurbitacins is the cause of their effects on microtubules. It can be suggested that cucurbitacins may suppress microtubules by indirectly affecting the microtubule-regulating proteins that are involved in microtubule dynamics.

In cell cycle analysis, reference drugs colchicine and vinblastine were shown to induce G2/M arrest, which agrees with the literature that they depolymerize microtubules or prevent tubulin assembly by binding to colchicine domain and vinca domain, respectively (Kavallaris, 2010; Wink, 2007; Wink & Schimmer, 2010). Colchicine and vinblastine alter the dynamic of mitotic spindles during mitosis, which triggers the cell cycle checkpoint and thus arrests the cell cycle at G2/M phase (Jordan & Wilson, 2004; Wink, 2016). The actin-binding agent latrunculin B induced G2/M arrest as well. Cdc25 has been reported to be involved in cell size monitoring via a checkpoint mechanism during mitosis (Coleman & Dunphy, 1994; Rupes et al., 2001; Russell & Nurse, 1986). Latrunclin B can dramatically alter cell morphology, which activates the checkpoint that linked to Cdc25 and thus block the cell cycle. Cucurbitacin B, E and I also showed the potential ability to arrest cell cycle at G2/M phase during the study (Fig. 6). These results consist with the findings from other studies (Deng et al., 2016; Duangmano et al., 2012; Yin et al., 2008), which further demonstrates their role as anti-mitotic agents. The concentrations of cucurbitacin B, E and I to arrest cell cycle were consistent with their cytotoxic concentration, suggesting that cucurbitacin B, E and I induce apoptosis mainly via cell cycle arrest. Cucurbitacins has been reported to induce G2/M arrest by decreasing cyclin A, cyclin B, cdc25C and increasing p21WAF1 (Chen et al., 2012). According to our previous findings, the alteration of microtubule dynamics could be a new explanation for their modes of action.

## Conclusions

Our study systematically investigated the roles of cucurbitacins in biological processes related to cytoskeletal microtubules and actin filaments. Our data suggest that cucurbitacin B, E and I interact with the cytoskeleton by mainly affecting actin filaments through depolymerization and aggregation, which provides evidence that actin may be one of the key targets of cucurbitacins. In addition, cucurbitacins altered mitotic spindles and induced G2/M arrest, indicating their potential role as anti-mitotic agents. These results allow a more comprehensive understanding of the changes of cancer cells responding to cucurbitacins. More studies at a molecular level are necessary to better understand these results and to use cucurbitacins in chemotherapy.

## Additional Information and Declarations

### Data Availability

The following information was supplied regarding data availability: Wang, Xiaojuan; Tanaka, Mine; Peixoto, Herbenya Silva; Wink, Michael (2016): Supplemental Files.zip. figshare. https://doi.org/10.6084/m9.figshare.4284572.v1.
